# *Strongyloides stercoralis *infection in a Finnish kennel

**DOI:** 10.1186/1751-0147-49-37

**Published:** 2007-12-12

**Authors:** Kati J Dillard, Seppo AM Saari, Marjukka Anttila

**Affiliations:** 1 Pathology Unit, Finnish Food Safety Authority Evira, Helsinki, Finland; 2 Department of Basic Veterinary Sciences, University of Helsinki, Helsinki, Finland

## Abstract

**Background:**

Intestinal threadworm *Strongyloides stercoralis *is a parasite of dog, cat and primates that occurs worldwide being most prevalent in tropical and subtropical countries. The adult parasitic worm is about 2 mm long and slender. It possesses both parasitic and free-living lifecycles. The parasitic worms are females. *Strongyloides stercoralis *infects the host via percutaneous, peroral or transmammary transmission in addition to autoinfection. Clinical disease varies from inapparent to severe enteritis and pneumonia. The diagnosis is based on demonstration of larvae in fresh faeces, which is best made by Baermann technique.

**Case presentation:**

*Strongyloides stercoralis *infection was diagnosed in autopsy in a 10-week-old puppy born and raised in a Finnish kennel. Prior to its sudden death, the puppy had suffered from gastrointestinal disturbance for three weeks. Subsequent sampling of the dogs in the kennel revealed that three adult dogs in the kennel were also infected.

**Conclusion:**

The present case shows that *S. stercoralis *can complete its life cycle and cause disease in dogs also in Northern Europe. Infection can be maintained also in a temperate climate and may become a chronic problem in a kennel environment. Infection may be underdiagnosed as Baermann technique is not routinely performed in small animal practice.

## Background

Species of *Strongyloides *are unique parasites in several respects. Many of them have two forms: a parasitic form consisting of partenogenetic females and a free-living form consisting of males and females that can live and reproduce in the soil outside the host. In addition, their life cycle can involve a process called autoinfection, i.e. they are able to multiply and complete its life cycle within a definitive host [1-7]. *Strongyloides *species of veterinary importance include a species infecting horse (*S. westerii*), cattle (*S. papillosus*) and swine (*S. ransomi*). These parasites are pathogens for young animals. In light infections, animals show no clinical signs. Young animals with heavy burdens may show acute diarrhoea, weakness, emaciation; even sudden death may occur [7]. *Strongyloides stercoralis *is a small thread-like nematode infecting dog, cat and primates including man. It occurs commonly in tropical and subtropical areas but may be found also in temperate areas [5,6]. According to recent textbook, it has been reported as a canine parasite from following European countries: Portugal, France, Poland, Ukraine, Romania, and Hungary [7]. To our best knowledge, it has not been reported previously in dogs in Northern Europe. However, some studies have shown that *S. stercoralis *is not exclusively a parasite of warmer climates. For example, it has been detected in arctic foxes (*Alopex lagopus*) in Greenland, where in certain areas 14 percent of arctic foxes studied has been infected [8]. This report is further evidence supporting that *S. stercoralis *may rather possess worldwide geographical distribution of as here we describe a case of infection associated with enteritis in a puppy, born and raised in Finland.

## Case presentation

### Clinical features

A 10-week-old Yorkshire terrier puppy was submitted to autopsy after three weeks of intermittent diarrhoea, vomiting and pain at defecation followed by sudden death. The clinical symptoms were first noted by the owner on the second day of arrival to the new home at the age of seven weeks. A foreign body was suspected and the puppy underwent explorative surgery. At this time the puppy was given 10 days of trimethoprime-sulphadiatzine medication (Ditrim^®^, Orion Pharma, Finland).

### Autopsy findings

**Figure 1:**
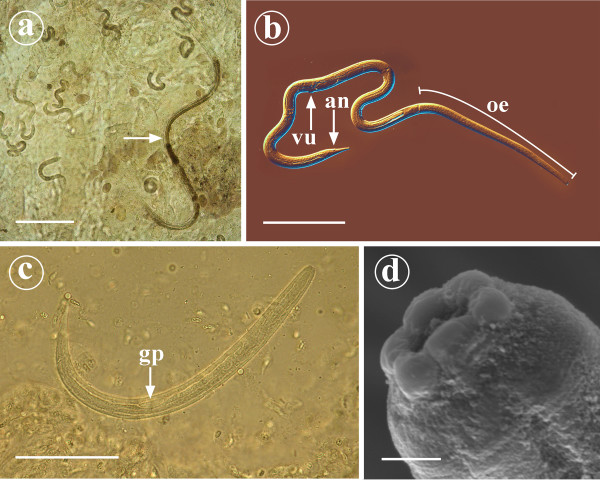
**Parasitological findings detected in 10-week-old Yorkshire terrier puppy suffering from *Strongyloides stercoralis *infection**. Figure 1a: In the intestinal scrapings of the duodenum numerous adult small female nematodes (arrow), larvae and ova were found. Scale bar = 200 μm. Figure 1b: Adult parasitic female possesses long cylindrical oesophagus (oe) that occupies the anterior third of the body. Vulva (vu) and anus (an) are located in the posterior third of the body and the tail is narrowly tapered. Scale bar = 200 μm. Figure 1c: First stage larva of *S. stercoralis*. Genital primordium (gp) is very prominent. Scale bar = 50 μm. Figure 1d: The anterior end of a parasitic female *S. stercoralis *as observed under SEM. Hexagonal oral opening is surrounded by six well-defined lips. Scale bar = 2 μm.

The nutritional condition was normal at necropsy. There was moderate oedema around the anus. In the duodenum and at the beginning of jejunum the mucosa was oedematous with moderate hyperaemia. The caudal small intestine and large intestine were moderately dilated with liquid contents. The colonic mucosa was dark red. Numerous small nematodes, larvae and ova were found in intestinal scrapings of the duodenum and also lesser amounts in other parts of the small intestine (Figure 1a).

### Parasitological studies

The adult nematodes were 2.0 – 2.5 mm long, up to 35 μm wide females with long cylindrical oesophagus that occupied the anterior third of the body (Figure 1b). Vulva was located in the posterior third of the body. The tail was narrowly tapered. The genital tract was paired and the uteri contained a small number of developing eggs. Larvae were the most abundant stage observed in mucosal scrapings. They were 200 – 250 μm long, with rhabditiform oesophagus and conspicuous genital primordium (Figure 1c). Some adult females were fixated, dehydrated, critical point dried and routinely processed for scanning electron microscopy (SEM). In SEM the females possessed hexagonal mouth surrounded by six clearly defined papillae (Figure 1d).

### Histopathology

**Figure 2:**
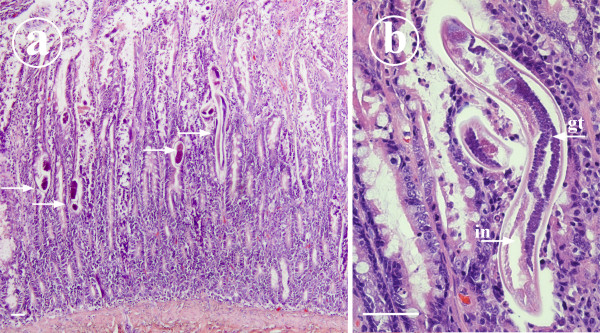
**Histopathology as seen in the duodenal mucosa of 10-week-old Yorkshire terrier puppy suffering from *S. stercoralis *infection**. Figure 2a: A micrograph to show numerous *S. stercoralis *larvae and ova (arrows) within the intestinal mucosa. In the mucosa there is moderate diffuse infiltration of lymphocytes and plasma cells. Haematoxylin-eosin stained histological section, scale bar = 50 μm. Figure 2b: A close-up micrograph with female *S. stercoralis*. The small size of the parasite, the relatively large intestine (in) and paired genital tract (gt) are readily seen in this longitudinally sectioned female. Haematoxylin-eosin stained histological section, scale bar = 50 μm.

Samples of all major organs were collected during necropsy of the puppy. Tissue samples were fixed in 10 per cent buffered formalin, routinely processed, embedded in paraffin, sectioned at 4 μm and stained with haematoxylin and eosin. Intestinal sections were also stained with Warthin Starry silver stain and Gram stain for bacteria. In the small intestine there were numerous intramucosal nematodes and larvae with moderate inflammatory cell infiltrate consisting of lymphocytes and plasma cells (Figure 2a and 2b). Adult nematodes possessed long muscular oesophagus, paired genital tract, platymyarian meromyarian musculature and an intestine composed of uninucleate cells. Occasional crypt abscesses with excess mucus were present. In the colon there was acute superficial necrotizing inflammation with numerous Gram+ bacteria on the surface. In addition there was large number of Gram-, silver staining spirochetes deep in the mucosa with no associated pathology. In the lungs there was multifocal moderate interstitial pneumonia. In the affected areas the alveolar septae were thickened and infiltrated by lymphocytes admixed with haemosiderin-laden macrophages. No larvae were found in the lungs. In the spleen and colon the lymphoid tissue was moderately depleted. No other lesions were found.

### Microbiological studies

Samples from intestine were cultured on blood agar for aerobic and anaerobic bacteria, on selective agars for *Campylobacter *sp. and *Yersinia *sp. and enriched for *Salmonella *sp. There was mixed bacterial growth with enterotoxigenic *Clostridium perfringens *type A predominating in the large intestine. Bacterial cultures were negative for *Salmonella *sp., *Campylobacter *sp. and *Yersinia *sp.

### Conditions in the kennel, sampling of the adult dogs and control of the infection in the kennel

All dogs were group-housed in heated, wooden dog-houses with attached grass runs. At parturition bitches were confined in a room with glazed tile floor and a wooden whelping box. The puppies born approximately at the same time were often mixed after weaning and they were housed in a room with glazed tile floor until they left to their new homes at the age of 7 weeks.

Faecal samples were collected from all the 41 dogs of various breeds in the breeder's kennel and from five other dogs that had been in contact with these dogs. The samples were stored refrigerated prior to analysis. Samples (5 g/dog) were pooled with 10, 10, 10, 11 and 5 samples per pool to examine the faeces as fresh as possible. The Baermann method was used [7]. In two pools a few larvae with morphology typical of *S. stercoralis *L1 stage were found. The 20 samples belonging to these pools were then processed individually using the same method. Three of these samples were positive. One of the adult dogs with positive faecal sample had been imported from the Netherlands three years ago and was housed with two other bitches also imported from the Netherlands at the same time. The two other positive dogs were adult bitches born in the kennel and housed together with a third dog. These six dogs were re-examined four weeks after treatment of all dogs with ivermectin (Ivomec^® ^Merial, France; 200 μg/kg, SC), and were found negative. Two of these positive bitches had recently had puppies. In order to reduce the environmental infective larval burden and the possibility to produce free living generations, several control measures aiming at cleaning and drying the environment were used in the kennel. Also deworming strategies of the kennel were revised: periodic treatments with fenbendazole were suggested and follow-up faecal examinations were advised.

## Discussion

*Strongyloides *infections are often moderate and asymptomatic, and disease occurs mainly in massively challenged neonates and nurslings. In dogs the severe infections involve pneumonia and watery to mucous diarrhoea. In this case there was marked parasitic infestation in the gut with clinical symptoms of at least 3 weeks' duration. The mild interstitial changes present in the lung at the time of necropsy may have been caused by migrating larvae, however, no parasites were found in the lung samples at the time of necropsy to confirm the etiology of the lesions. The morphological characteristics of the adult nematodes and L1 larvae were typical for *S. stercoralis *which can be differentiated from other *Strongyloides *species on the basis of the hexagonal shape of the mouth and tail of the adult female [3]. In tissue sections the adult females and larvae are typically found within the crypts of small intestine [9]. In symptomatic infected dogs, gross intestinal changes range from congestion of mucosal surface with abnormal abundance of mucus in the lumen, to confluent ulceration. In severe infection, large numbers of parasites are present in the intestinal wall and there may be pulmonary haemorrhage due to large numbers of migrating larvae [4,5].

The infection is not easily recognized by routine methods. The larval output is irregular and may be low in adults [10]. The larvae passed in faeces become easily crenated and unrecognizable when saturated salt solutions are used. Although, faecal flotation with zinc sulphate can yield identifiable larvae, direct smears of faecal sample or preferably the Baermann technique are recommended methods for detecting *S. stercoralis *larvae [2,4]. The apparatus needed for the technique consists of a glass funnel held in a retort stand, a rubber tube constricted with a clip attached to the bottom of the funnel and a sieve or a small bag made from double layered gauze. The faecal sample is placed in the sieve in the wide part of the funnel, and the funnel is filled with water until the faecal sample is immersed. The apparatus is left at room temperature for several hours during which the larvae migrate out of the faeces and through the sieve and sediment at the bottom of the funnel. The sediment can be collected and examined under microscope [7].

Overgrowth of *C. perfringens *in the gut was the likely cause of death in this puppy. Little is known about the pathogenesis of clostridial infections in dog. It is still uncertain whether *C. perfringens *is a primary or secondary cause of diarrhoea in dogs, but there are published reports in which strains of *C. perfringens *type A have been associated with fatal diarrhoea [11]. In these cases a superficial necrotizing inflammation is typically found in the intestine. Bacterial overgrowth in the small intestine is commonly found in people suffering from strongyloidosis [12]. In our case it seems likely that the bacterial overgrowth occurred secondary to the parasitic infestation of the intestinal mucosa.

It seems likely that the infection in the kennel was maintained due to continual presence of new puppies. The dogs of the kennel had an access to grass runs, but as *S. stercoralis *is distributed in humid tropical and subtropical regions, its capability to complete free living life cycle, its fertility and lifespan are probably all affected by temperature and humidity. Thus, it is unlikely that the contaminated grass runs played a significant role in the epidemiology of the present case. In addition the infected puppy had been raised indoors. The infection can be maintained and it may be difficult to control in a kennels due to transmission from dam to pups via milk. Overcrowding and poor hygiene in a kennel are predisposing factors [6,13].

**Figure 3:**
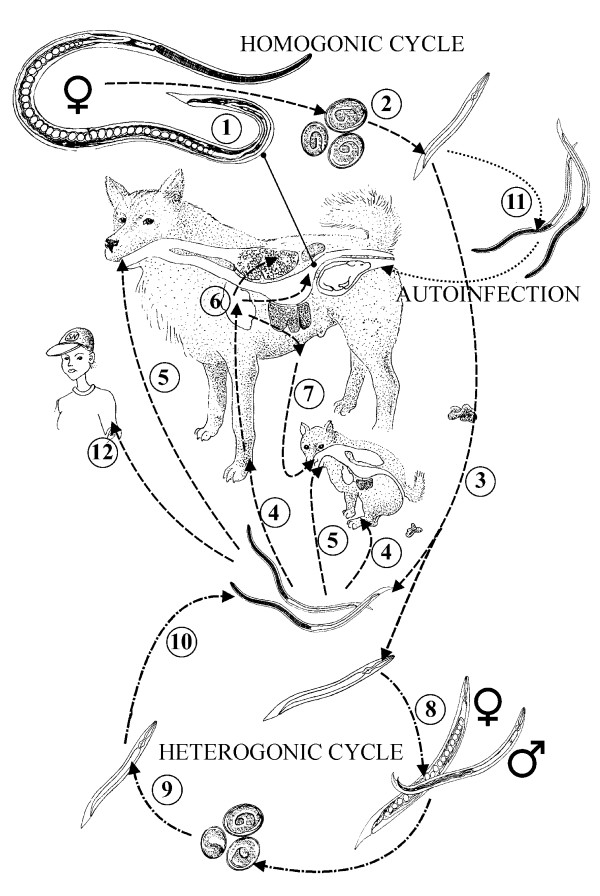
**Life cycle of *S. stercoralis***. All adult parasites are partenogenetic females (1) that reside in the crypts of small intestine. Their embryonated eggs (2) hatch in the crypts or the intestinal lumen. The L1 rhabditiform larvae are voided in faeces (3) and give a rise either to L3 infective filariform larvae (L3i) or develop to free living adult nematodes depending on environmental conditions. The L3i develops no further if it does not gain access to new host. The L3i enters the dog percutaneously (4) perorally(5). The larvae migrate to the small intestine and molt first to L4 and then tothe parthenogenic adult female(6). The puppies can be infected via milk if the bitch has migrating L3 larvae (7). Transplacental infection does not occur. This parasitic life cycle of *S. stercoralis *is referred as homogonic life cycle. If environmental conditions are optimal, an alternative route of life cycle (heterogonic life cycle) can take place. Non-infective rhabditiform larvae develop to free-living adult male and female worms (8) that produce eggs. Non-infectious rhabditiform larvae (9) hatching from the eggs will develop to L3i (10). During passage through the host intestinal tract, rhabditiform larvae may rapidly undergo molts into L3i. These larvae can penetrate through the wall of large intestine or perianal skin of the host resulting in migration ending in the small intestine (11). The process is called autoinfection and it is favoured especially in neonatal or immunocompromized hosts. The canine strains of *S. stercoralis *have been known to infect humans (12). The life cycle was drawn based on the information obtained from following references: [2,4,16–18].

Currently it is not known whether a latent infection is reactivated during pregnancy thus making it easier for the parasite to find new susceptible hosts. There are two hypotheses how reactivation could happen: either the postreproductive female worms in the mucosal crypts, or the parenteral third stage arrested larvae are reactivated as a result of a change in the hormonal status due to pregnancy and/or immune status due to corticosteroid treatment. These two hypotheses have been studied by Mansfield and others [14]. They found that some postreproductive females were long-lived and were capable of producing larvae, when the host was treated with corticosteroids. They also found parenteral *Strongyloides *larvae two months after infection, but were not able to show migration of these larvae to the intestine [14]. The complex life cycle of *S. stercoralis *is presented in details in Figure 3.

Ivermectin treatment is effective in removing the adult parasites from the intestinal tract but not larvae from parenteral sites [4,15]. However, it is not an approved drug and may cause serious side effects in some dogs. Thus, fenbendazole is usually the drug of choice in treatment of *S. stercoralis *infection [4].

*Strongyloides stercoralis *is a zoonotic parasite, and even though natural transmission from dog to man has been only rarely reported, the potential danger should always be taken into account when dealing with infected dogs [5,6]. Clinical signs seen in human infections resemble those observed in dogs, i.e. the majority of the infections are either asymptomatic or mild and non-specific. However, immunodeficient patients are more susceptible. Their impaired immune reactions are incapable to control the vicious circle of continuous autoinfections. This may lead to hyperinfection and disseminated strongyloidosis, which may be fatal [3,6].

In conclusion, it is evident that *Strongyloides stercoralis *can complete its life cycle and cause serious disease in dogs in Northern Europe. As Baermann technique is not routinely performed in small animal practice, *Strongyloides *infection may actually be more common in countries of temperate climate than previously thought.

## Competing interests

The author(s) declare that they have no competing interests.

## Authors' contributions

KD was responsible for the necropsy, parasitological examination and identification of the *Strongyloides stercoralis *infection. SS was responsible for taking micrographs and the morphological description and scanning electron microscopy of the parasite. KD and MA were responsible for the histological examination, interpretation of the bacteriological data, and collecting data and samples from the kennel. All authors have been involved in drafting the manuscript. All authors have given final approval of the manuscript.
